# Supplementary material for: Precise measurements of chromatin diffusion dynamics by modeling using Gaussian processes
Source: Nat Commun. 2021 Oct 26;12:6184. doi: 10.1038/s41467-021-26466-7 (PMC8548522; doi:10.1038/s41467-021-26466-7)
Supplement: Supplementary file 1 — Supplementary Information [file 41467_2021_26466_MOESM1_ESM.pdf]

Precise measurements of chromatin diffusion dynamics by modeling using Gaussian processes

Guilherme M. Oliveira[1, *], Attila Oravecz[1], Dominique Kobi[1], Manon Maroquenne[1], Kerstin Bystricky[2], Tom Sexton[1,*], and Nacho Molina[1,*]

[1]Institute of Genetics and Molecular and Cellular Biology (IGBMC)
CNRS UMR7104, INSERM U1258, University of Strasbourg, Illkirch, France
[2]Molecular Cellular and Developmental Biology unit (MCD), Centre de Biologie Integrative (CBI)
UPS, CNRS, 31062 Toulouse, France
[*]Equal contribution and corresponding authors: molinan@igbmc.fr, sexton@igbmc.fr, ,
monteirg@igbmc.fr

Supplementary Table

Primer Name	Sequence
GAPDH S	5'- CATCACTGCCACCCCAGAAGACTG
GAPDH R	5'- ATGCCAGTGAGCTTCCCGTTCAG
Nanog S	5'- AGCAGAAGTACCTCAGCCTC
Nanog R	5'- CCGCTTGCACTTCATCCTTT
Hoxa1 S	5'- CACCAAGAAGCCTGTCGTTC
Hoxa1 R	5'- CGTAGCCGTACTCTCCAACT
Hoxa4 S	5'- GGTGTACCCCTGGATGAAGA
Hoxa4 R	5'- AAGACTTGCTGCCGGGTATA
Hoxa7 S	5'- AAGCCAGTTTCCGCATCTAC
Hoxa7 R	5'- CTTCTCCAGTTCCAGCGTCT
Hoxa9 S	5'- CAATGCCGAGAATGAGAGCG
Hoxa9 R	5'- CTGGTGTTTTGTGTAGGGGC
Hoxa13 S	5'- GCCAAATGTACTGCCCCAAA
Hoxa13 R	5'- CTATAGGAGCTGGCGTCTGA

Supplementary Table 1: Primers used for RT-qPCR.

Supplementary Figures

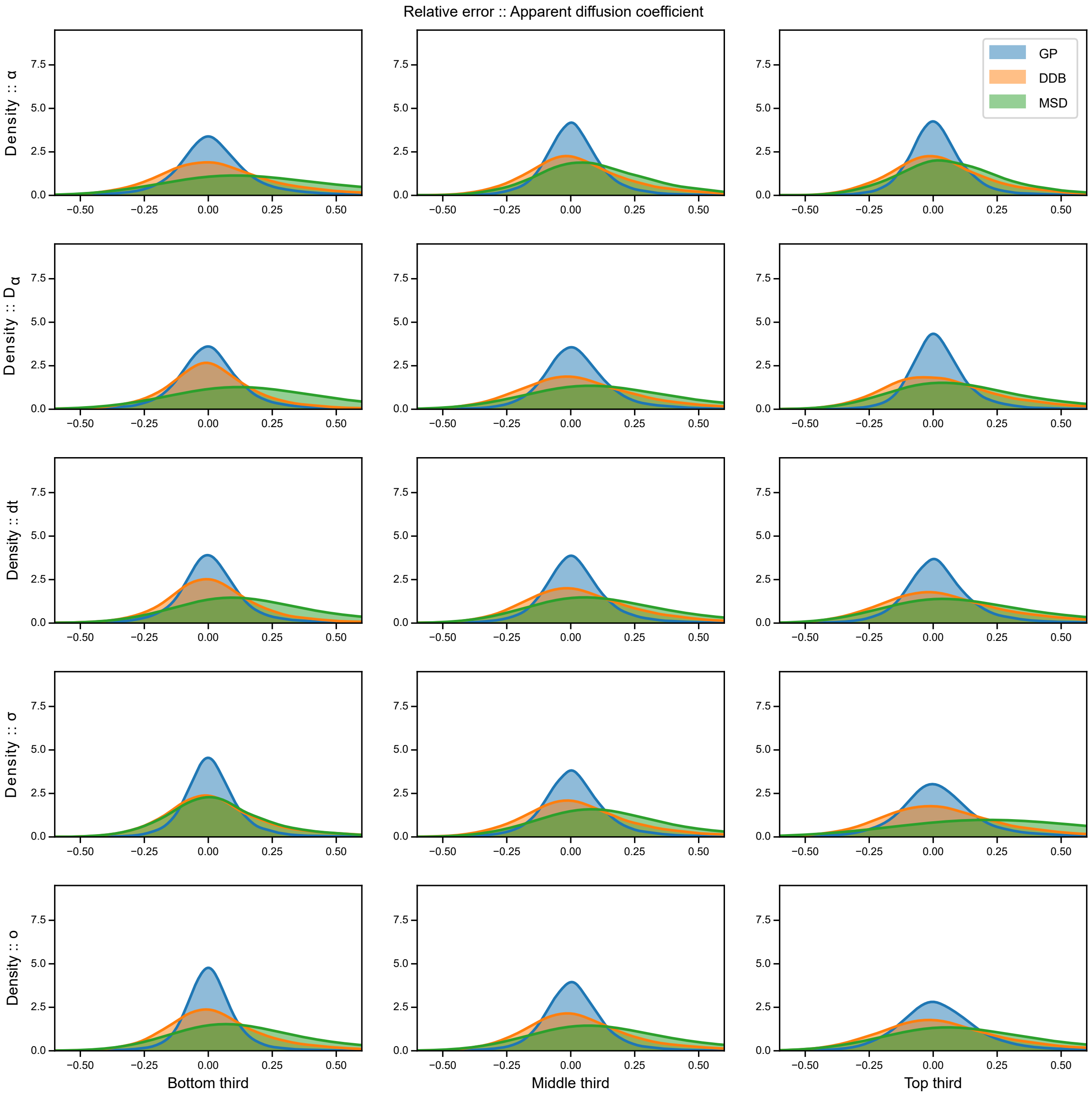

Supplementary Figure 1: Relative errors of inferred apparent diffusion coefficient. D_α values were estimated from simulated trajectories of 512 steps using randomly chosen parameters from a uniform distribution in the range $0.01 < D_\alpha < 1.5$, $0.01 < \alpha < 1.9$, $0 < dt < 5$, $0.001 < \sigma < 1.0$ and $0.0 < o < 0.8$, where dt is the time step, σ represents the localization error and o the occlusion ratio. To determine how GP-FBM performs in relation to MSD and DDB and assess how the performance depends on the parameter values, we simulate 50000 trajectories and bin parameters individually into thirds of their total range, whilst keeping full range for the others. As we can see, GP-FBM (in blue) outperforms MSD (in green) and DDB (in orange) in every case.

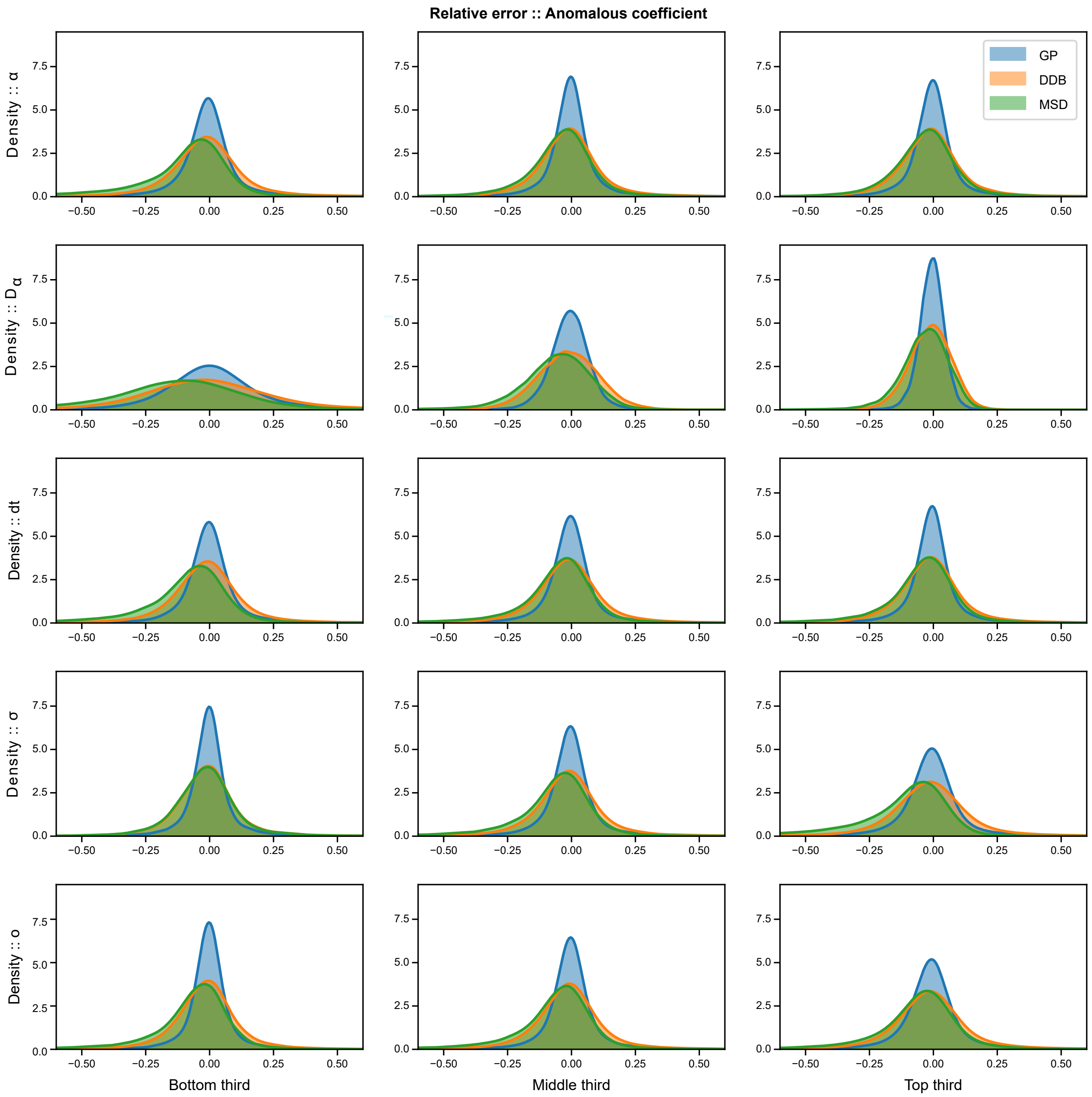

Supplementary Figure 2: Relative errors of inferred anomalous coefficients. α values were estimated from simulated trajectories of 512 steps using randomly chosen parameters from a uniform distribution in the range $0.01 < D_\alpha < 1.5$, $0.01 < \alpha < 1.9$, $0 < dt < 5$, $0.001 < \sigma < 1.0$ and $0.0 < o < 0.8$, where dt is the time step, σ represents the localization error and o the occlusion ratio. To determine how GP-FBM performs in relation to MSD and DDB and assess how the performance depends on the parameter values, we simulate 50000 trajectories and bin parameters individually into thirds of their total range, whilst keeping full range for the others. As we can see, GP-FBM (in blue) outperforms MSD (in green) and DDB (in orange) in every case.

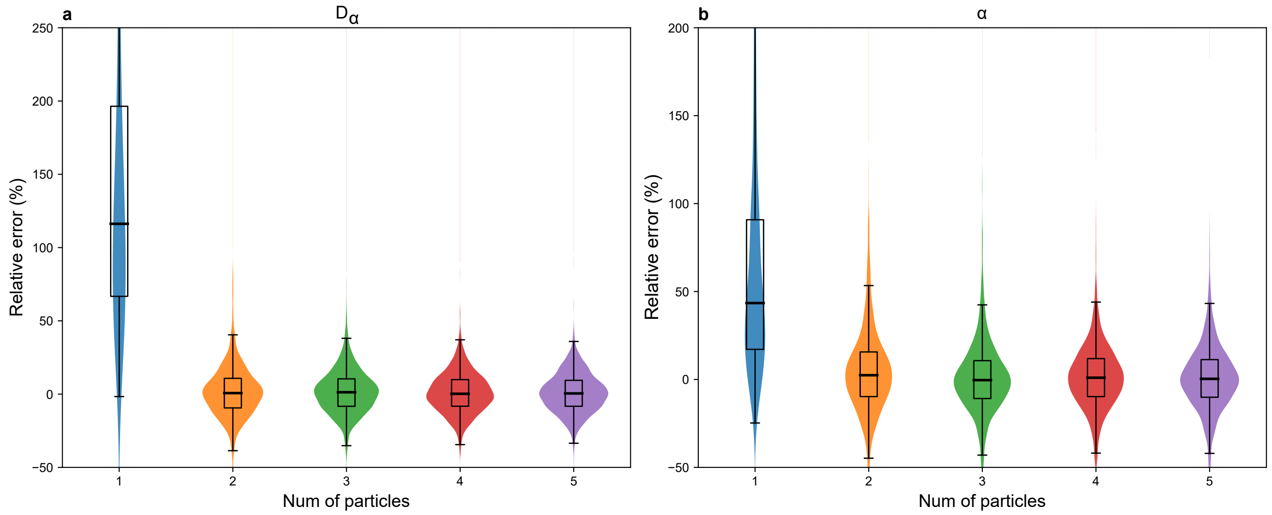

Supplementary Figure 3: Accuracy gain with number of particles when correcting for substrate movement. Relative errors of inferred D_α and α for 7680 simulated trajectories with introduced substrate movement, that is, 512 repetitions per box. Boxes represent the interquartile range and whiskers 95% of the data. Medians are shown as solid lines inside the boxes. Precision increases with greater particle numbers, but nearly all correction is essentially provided from two particles.

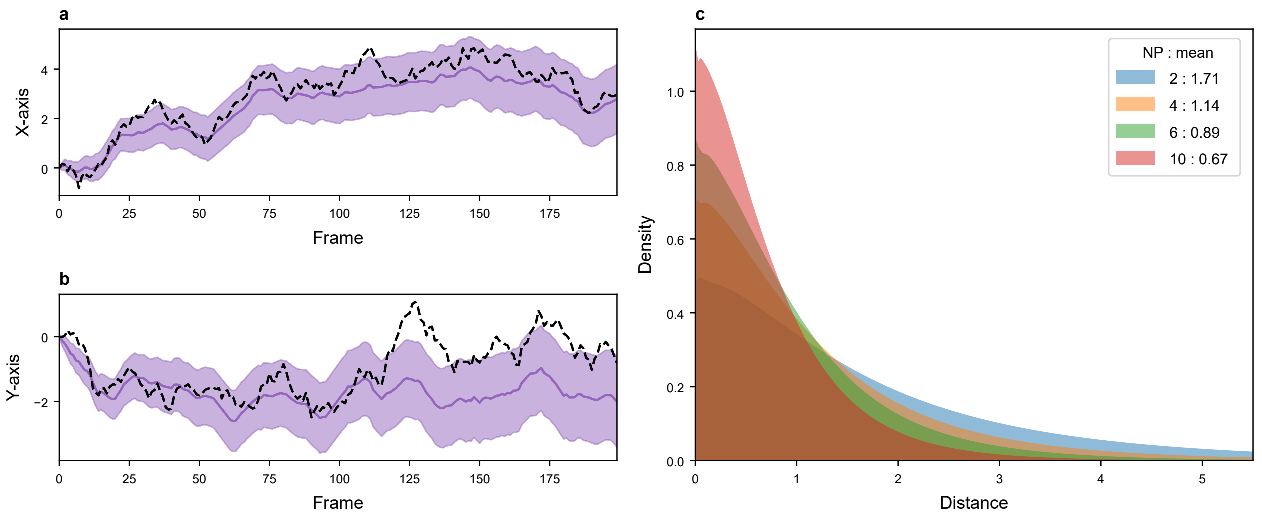

Supplementary Figure 4: Estimating background movement from tracked particles. (a,b) Black dashed lines correspond to simulated substrate movement, while purple line is the path estimated for the substrate using GP-FBM. Shaded area correspond to one standard deviation. (c) Localization error distribution in pixel units calculated between substrate simulated and estimated trajectories for systems with 2, 4, 6 and 10 particles.

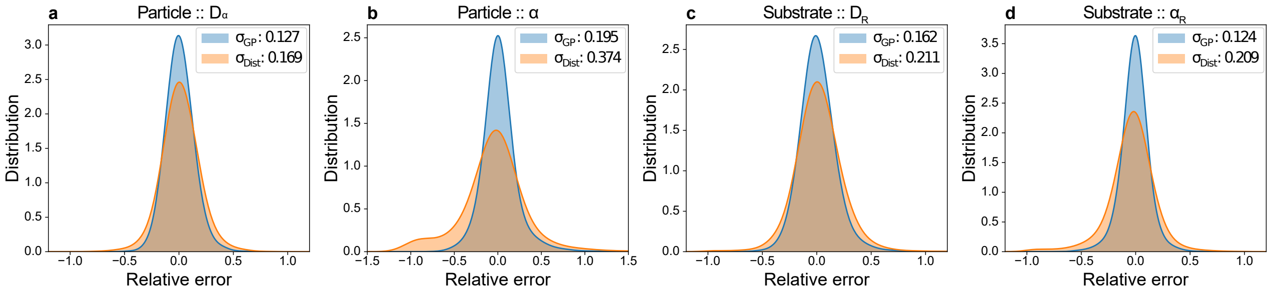

Supplementary Figure 5: Bench-marking GP-FBM and DDB approaches for inference of diffusion properties with substrate movement A set of 10,000 trajectories of two particles with substrate movement were generated. Simulated values were inferred using GP-FBM (in blue) and DDB (in orange) and relative errors of D_α and α were plotted for particles (a,b) and substrate (c,r). σ-values are one standard deviation.

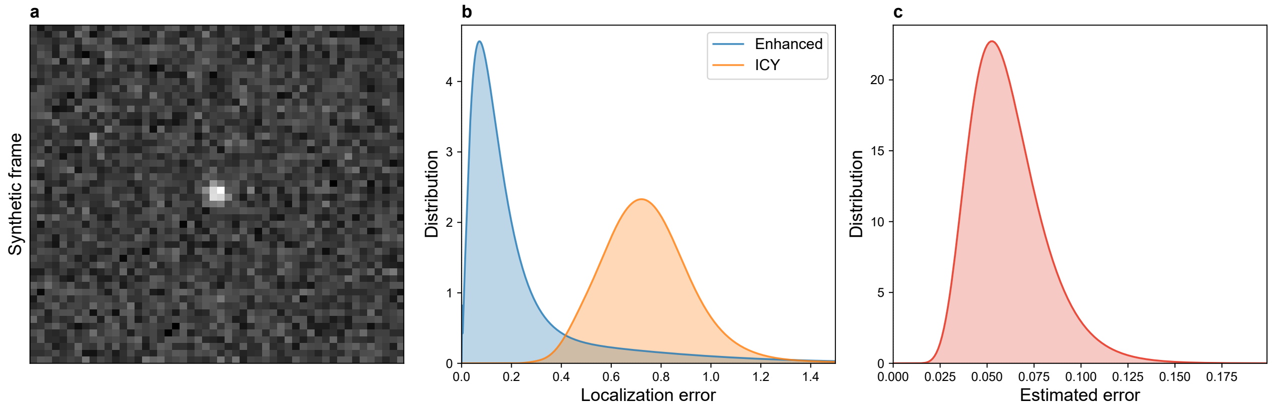

Supplementary Figure 6: Verifying localization enhancement algorithm via synthetic movie. To test this method, we generated a synthetic movie with 500 frames for a single particle trajectory. The spot was generated using a 2D symmetric Gaussian shape with deviation 1, background signal was set to 100 and spot intensity to 200. Signal noise was generated in the form of a Poisson distribution by taking original signal as mean. In (a) we have a frame of such a synthetic movie. (b) Spots were tracked with ICY and further enhanced as described in Methods. Distribution of localization errors were calculated using simulated trajectories as reference. (c) Distribution of localization error standard deviations, computed per spot with MCMC.

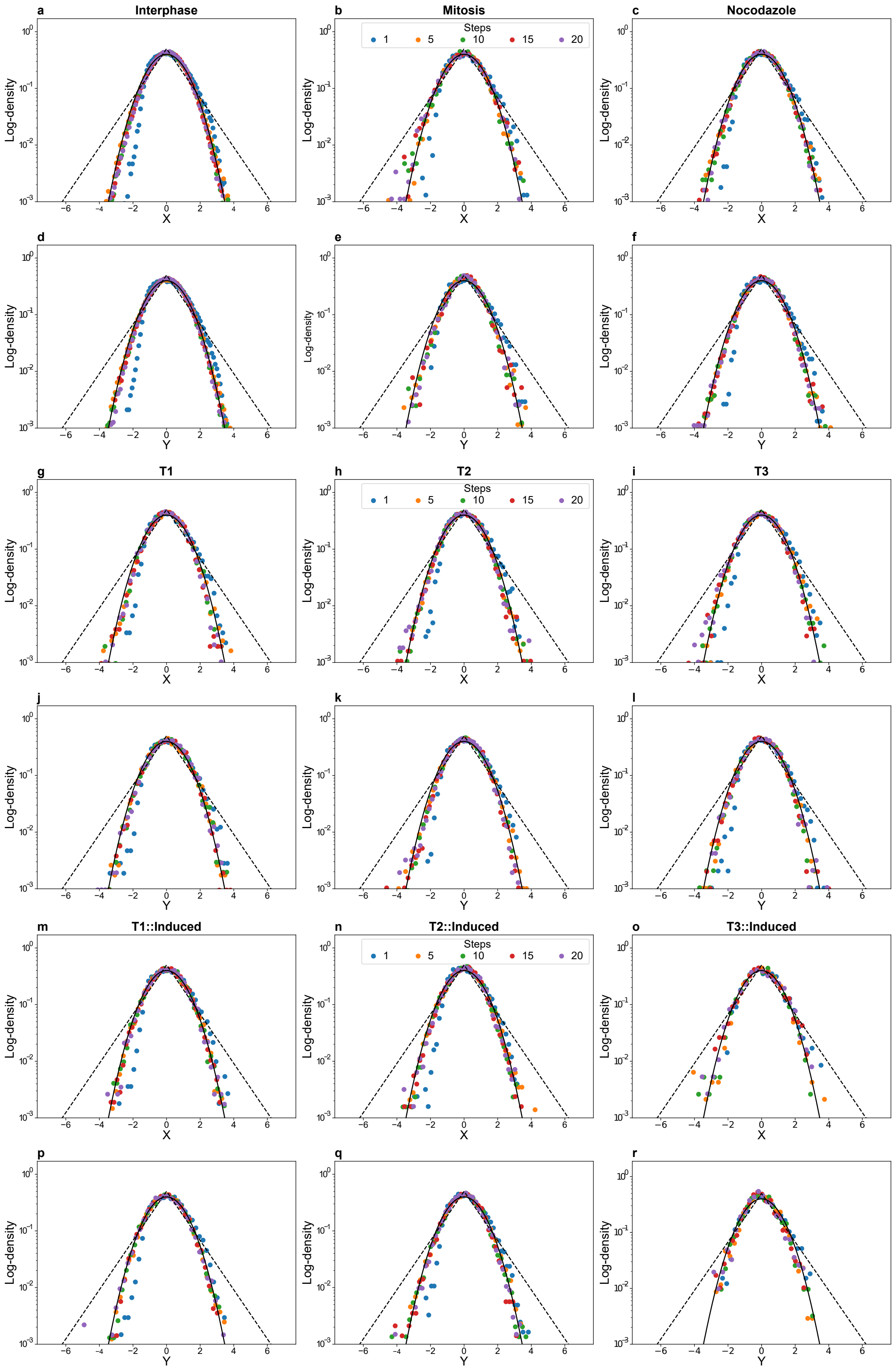

Supplementary Figure 7: Gaussianity and self-similarity test on experimental trajectories. Displacements were calculated and grouped for different time steps for all cell lines used in this manuscript and compared against standard Gaussian and Laplacian distributions. All displacement values for a given time step δ were normalized by $2D_\alpha\delta^\alpha$, where D_α and α were inferred from the corresponding trajectory. Experimental results show normally distributed and self-similar displacements over the time scales of our experiments, compatible with the FBM model.

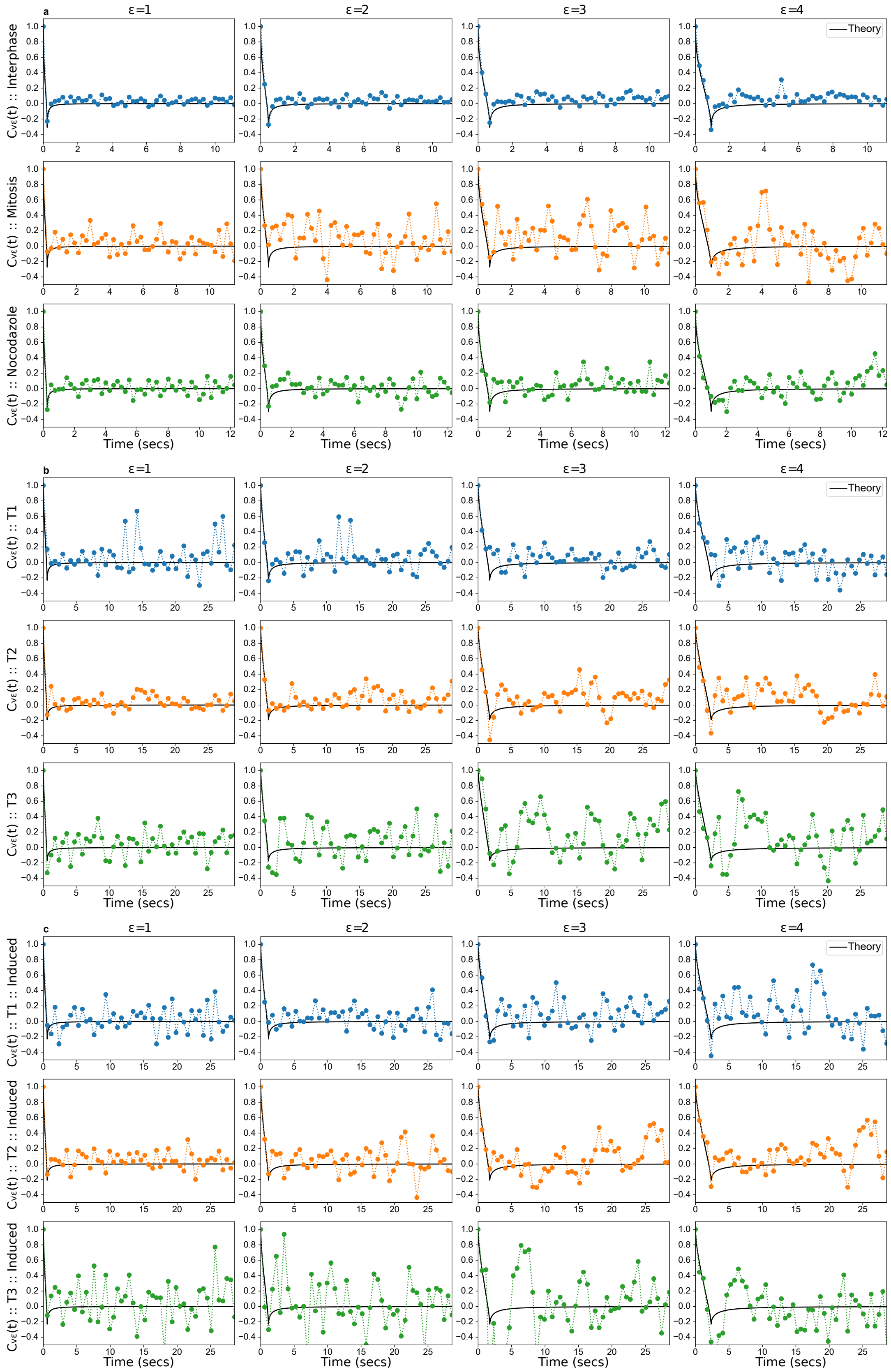

Supplementary Figure 8: Chromatin loci movement presents FBM velocity autocorrelation. Velocity autocorrelation functions were calculated using equation 9 and grouped per cell line in order to obtain smoother curves. Final results are compared to theoretical FBM curves given by equation 10, where average cell line anomalous coefficient was used.

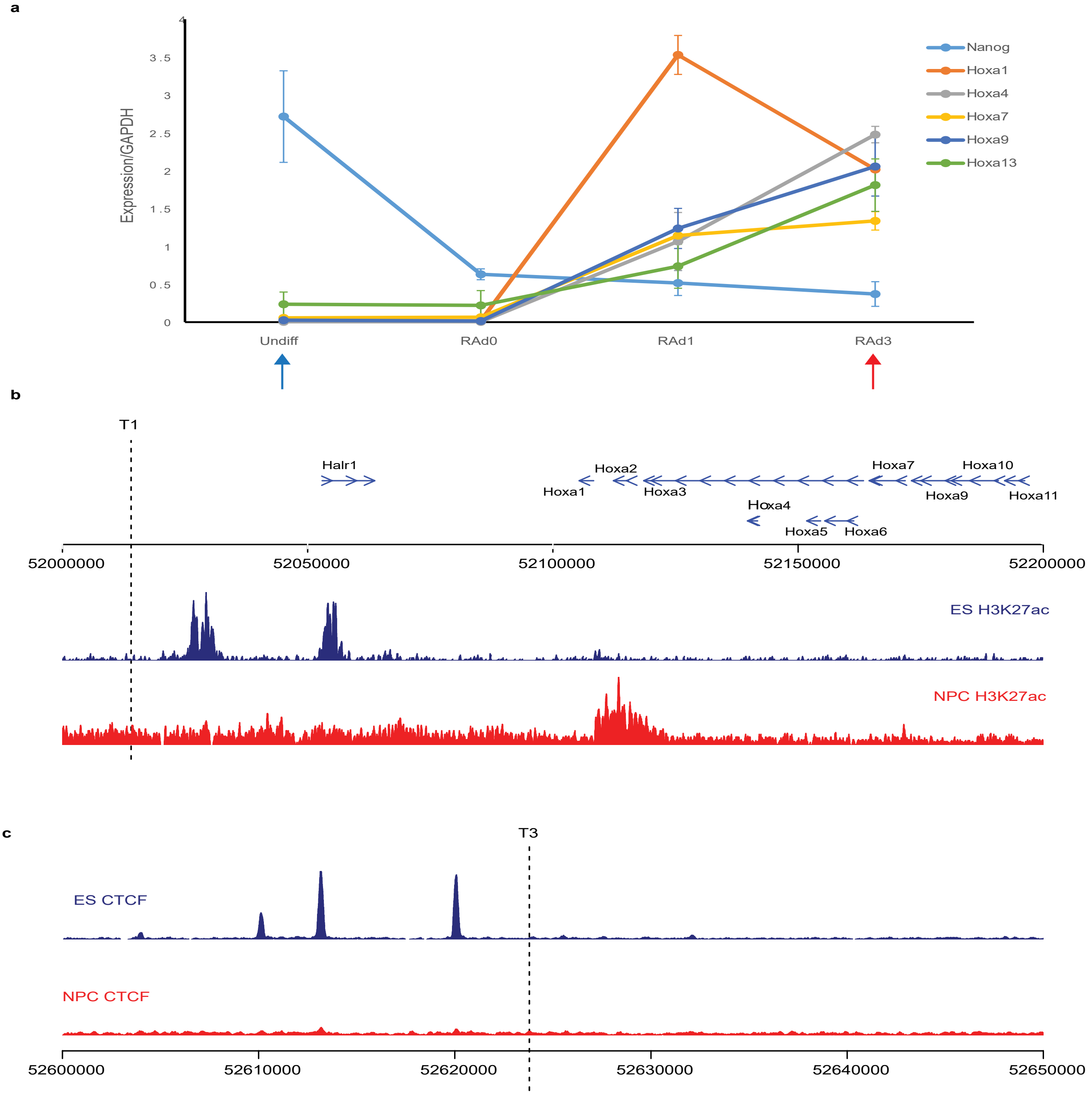

Supplementary Figure 9: Comparison between protein levels before and after differentiation. (a) RT-qPCR results, showing expression of Nanog and HoxA genes, normalized to GAPDH, in ES ANCHOR cells before and at different stages of induction by retinoic acid. The graph shows the average results over triplicate experiments. The error bars represent standard deviations. The blue and red arrows indicate the timepoints at which microscopy is performed, as in Fig. 4. (b) Gene positions and H3K27ac ChIP-seq tracks in ES (blue) and neuronal precursor cells (red) are plotted for a 200 kb window containing the HoxA cluster and the position of ANCHOR label T1. T1 is <15 kb from an ES-specific putative enhancer. (c) CTCF ChIP-seq tracks in ES (blue) and neuronal precursor cells (red) are plotted for a 50 kb window flanking the ANCHOR label T3. T3 is very close to ES-specific CTCF sites.

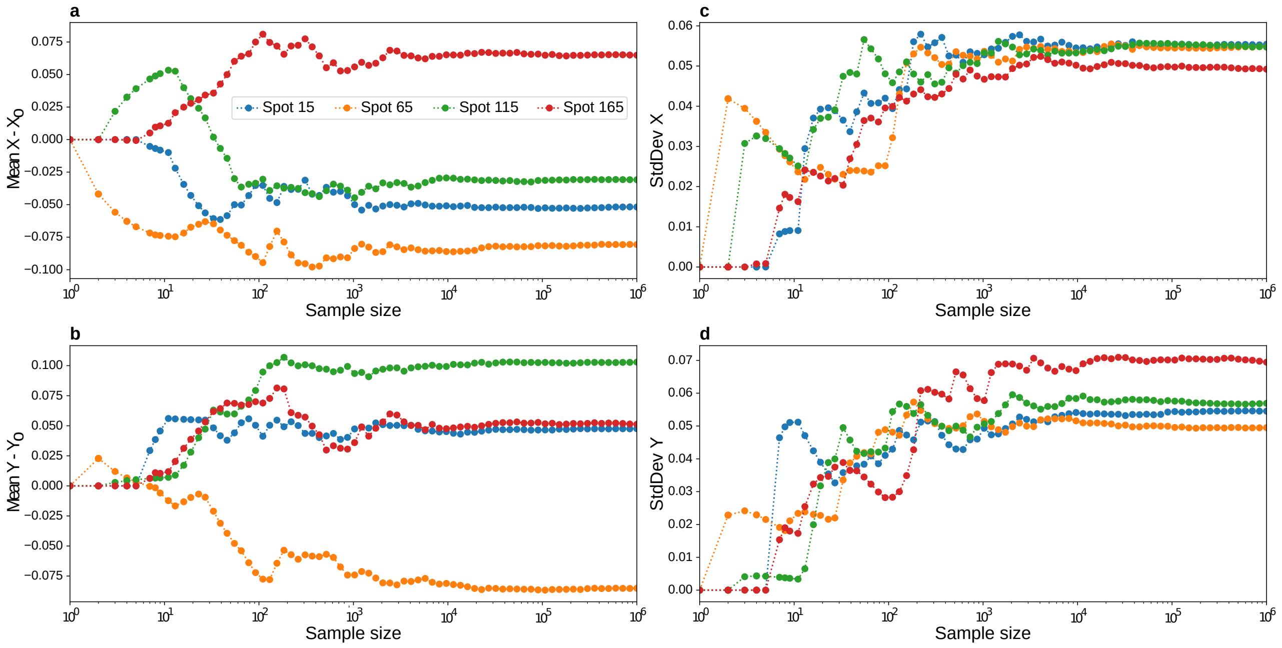

Supplementary Figure 10: MCMC sampler performance on spot localization of real movie. Spots are assumed to have a bell shape as describe in equation 4 and, initially, parameters are optimized via Nelder-Mead algorithm [3]. Then, we sample these parameters using Metropolis-Hastings sampler with acceptance ratio around 20%. (a,b) Evolution of the center of mass position in X and Y axis with increasing sample size for several real spots in a movie. (c,d) Estimate localization error with sample size. For the sake of efficiency, parameters were estimated using samples with 10^4 point for all movies presented in this manuscript.

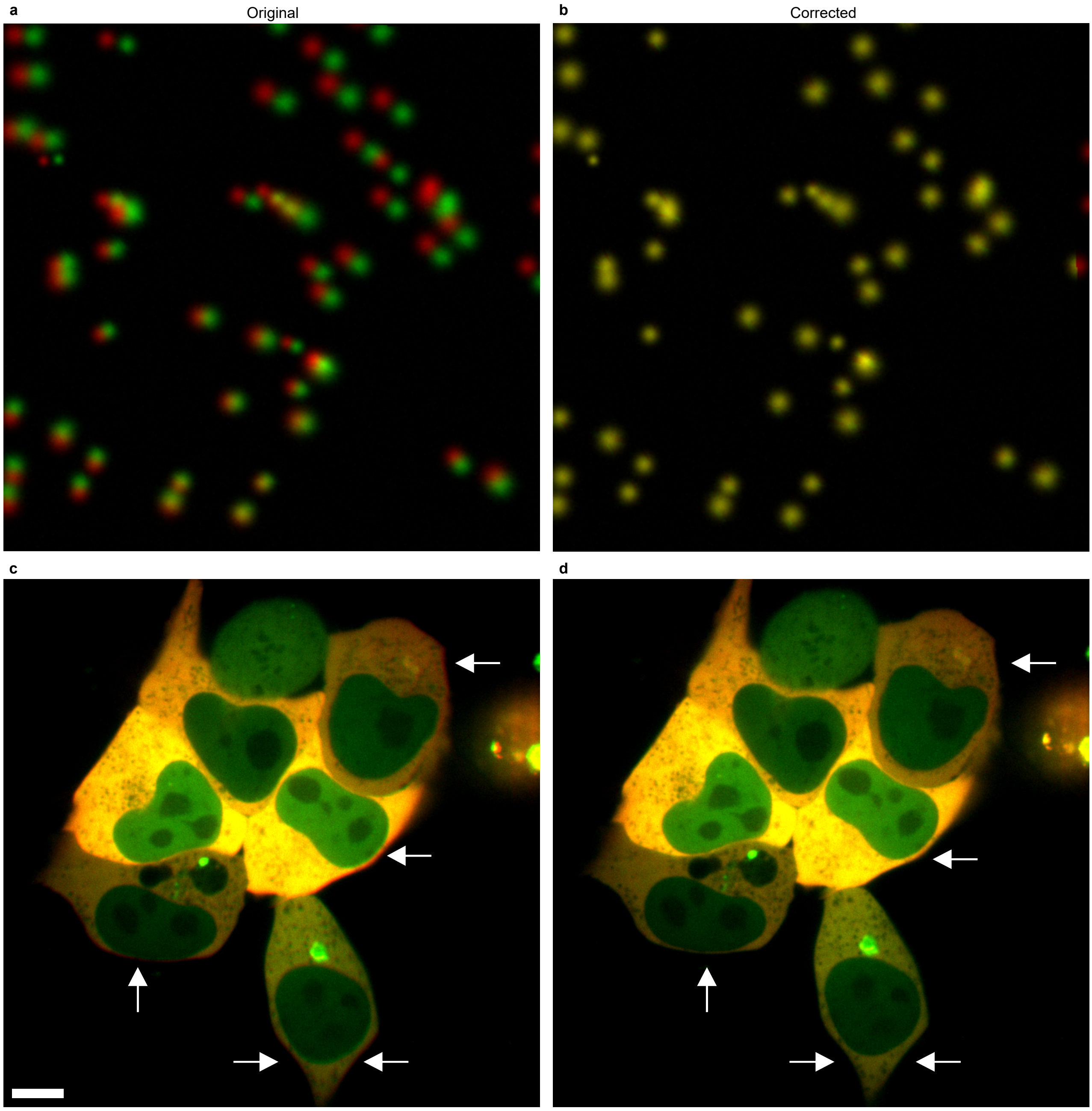

Supplementary Figure 11: Examples of misaligned channels before and after correction. (a,b) Simulated spots in which we exaggerate effects of camera misalignment. (c,d) White arrows point to regions with pronounced effects caused by chromatic aberrations. After alignment algorithm is deployed, both channels are properly superposed. Scale bar equals 10 μm and applies to all images.

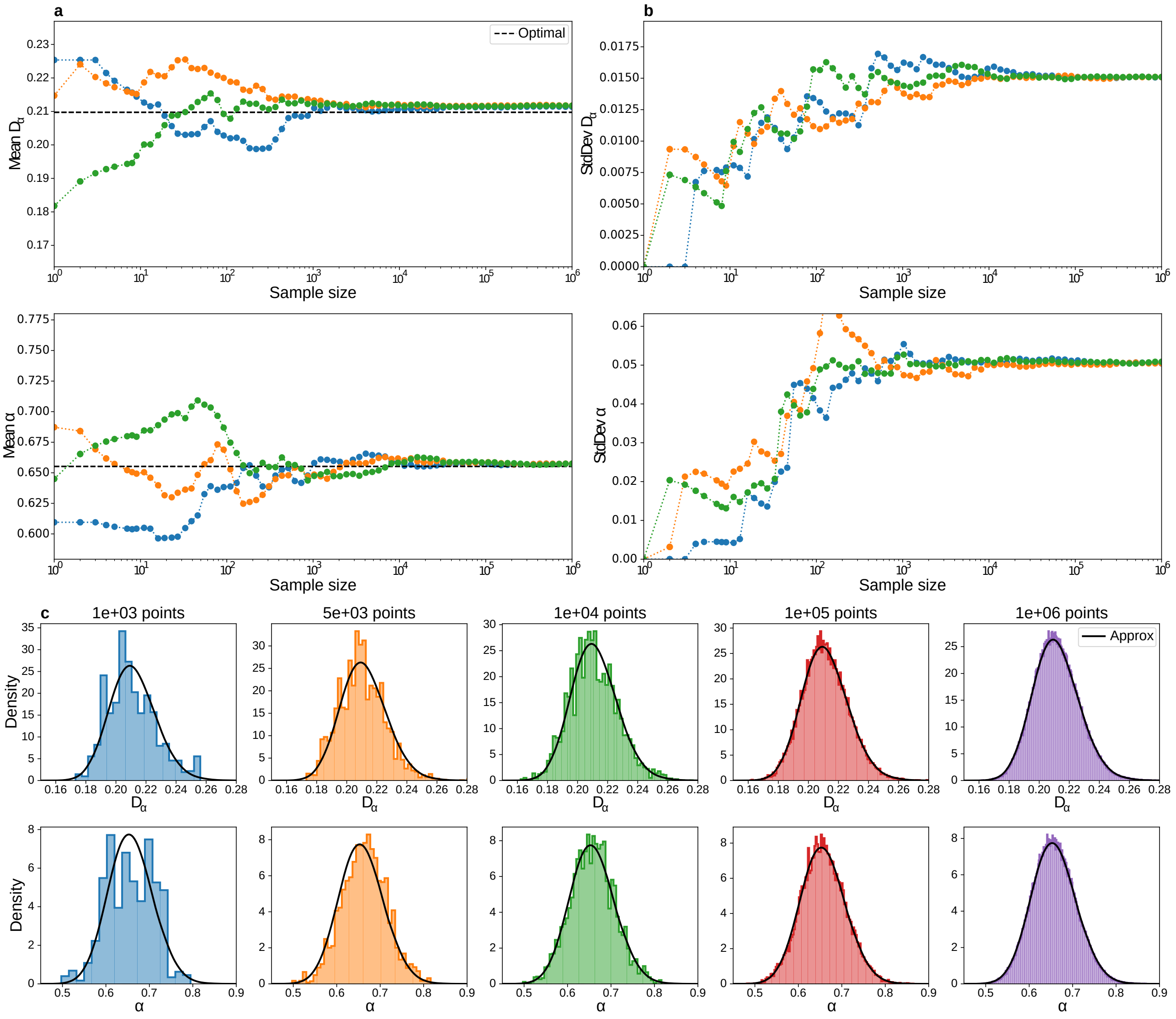

Supplementary Figure 12: Sampler performance on a real trajectory. Maximum posterior estimates for the apparent diffusion and the anomalous coefficient are calculated using the NM-Simplex method [3] due to its efficiency. To assess the inference uncertainty of these parameters we sample the posterior distribution using the Metropolis-Hastings algorithm [1, 2]. Starting from the optimal result, MH algorithm runs for a few thousand steps to self-calibrate to an acceptance ratio of about 20%and, after that, samples are collected. We show the evolution of the mean estimates (a) and their standard deviation (b) with sample size for a real trajectory of about 200 frames. In (c) we snapshot the probability density function generated for various sample sizes. Due to performance arguments, GP-Tool has 10^4 sized samples as default. Using a 11th Gen Intel(R) Core(TM) i7-11700 at ~4.8GHz on WSL 2 (Win 10), 10^4 points take about 10 seconds, whilst 10^6 take about 1000 seconds.

References

[1] Christophe Andrieu, Nando De Freitas, Arnaud Doucet, and Michael I Jordan. An introduction to MCMC for machine learning. *Machine learning*, 50(1):5–43, 2003.

[2] Kevin P Murphy. *Machine learning: a probabilistic perspective.* MIT press, 2012.

[3] John A Nelder and Roger Mead. A Simplex Method for Function Minimization. *The Computer Journal*, 7(4):308–313, 1 1965.

Supplementary information: GP-Tool

Installation

GP-Tool was tested in Linux, Windows and Mac OSX. It can be easily compiled under these systems given that compiling tools (GCC, MSVC or XCode compatible with standard c++17), OpenGL 4+, CMake and Git are already preinstalled and available. To clone the repository along with all necessary sub-modules use

```
git clone --recurse-submodules -j4 https://github.com/guilmont/GP-Tool.git
```

Linux and Mac OSX

Once cloning is completed, create a build folder and load ccmake as follows

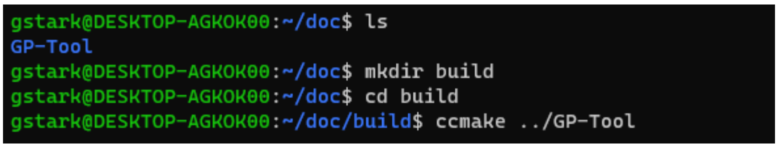

which will open ccmake. Type "c" to configure. You shall see

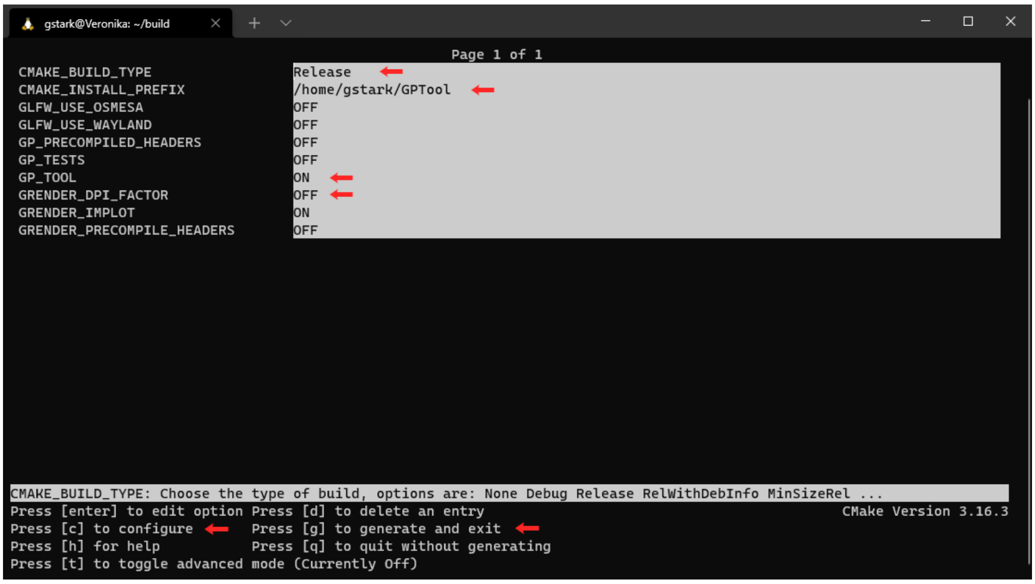

The relevant options to be addressed are (red arrows):

1. `CMAKE_BUILD_TYPE`: Conventionally should be set to "Release", unless debugging features are needed;

2. `CMAKE_INSTALL_PREFIX`: Set to the path in which GP-Tool shall be installed;

3. `GP_TOOL`: If "ON", the graphical user interface will be compiled and installed to installation prefix. This graphical interface will be installed only in Release mode;

4. `GRENDER_DPI_FACTOR`: If "ON", the size of all widgets will be scaled to compensate for high dpi monitors.

Once all the options are set accordingly, press "c" to reconfigure and "g" to generate. For Linux and Mac OSX, run:

```
make -j install
```

everything should be compiled and installed at the set installation prefix.

Windows

If the user plans to install GP-Tool from source, I would advice to install "cmake-gui.exe". Once you open it, set the source code address and where you would like to build the project. After that, click the button "Configure" at the bottom and specify the generator (I'm using 2019) and platform x64. Click "Finish". You will see something like this

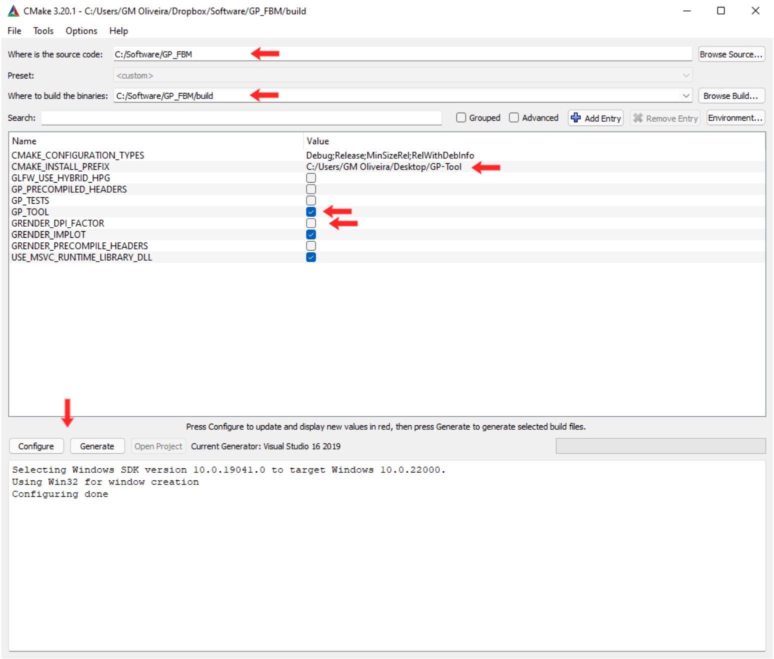

Several option will be prompted, but relevant ones are (red arrows):

1. `CMAKE_INSTALL_PREFIX`: Set to the path in which GP-Tool shall be installed;
2. `GP_TOOL`: If "ON", the graphical user interface will be compiled and installed to installation prefix;
3. `GRENDER_DPI_FACTOR`: If "ON", the size of all widgets will be scaled to compensate for high dpi monitors.

Then reconfigure and generate the configuration files by clicking in "Generate". Once configured, open the console and navigate to the build directory and type:

```
cmake.exe --build . --config Release -j --target install
```

Once the project is built, GP-Tool will be installed at the prefixed location. The user can also build the project in other configurations, however only libraries will be installed, that is, no graphical interface.

Plugins

GP-Tool is split into several interrelated plugins that work in synchrony. These are responsible by opening movies and handling their metadata, loading tracked trajectories in multiple formats, applying GP-FBM and correcting multi-channeled movies alignment. In the next few section*s, we are going to learn how to use each plugin in detail and how to save the final results of the analysis.

Movie

Once GP-Tool is opened, the user can load tiff movies via *Files > Open movies...* or by the key combination “Ctrl+O”. There are 3 formats of metadata that GP-Tool can parse: basic ImageJ, extended ImageJ and OME. GP-Tool can also handle movies compressed via LZW algorithm.

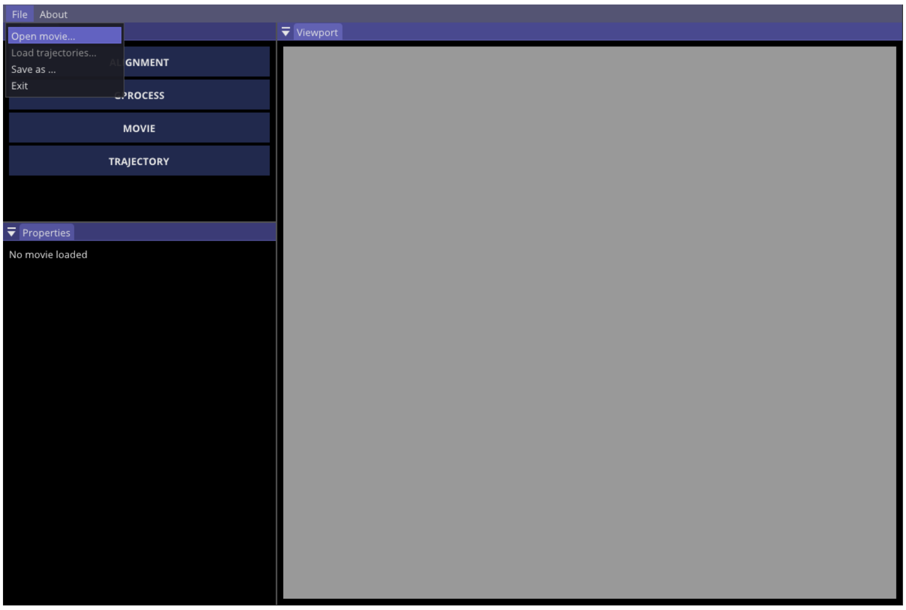

This selection will open the following file dialog, where the user will be able to navigate until the desired movie. For convenience, we can write or paste the address directly at the path box (red arrow) or drag the file/folder into GP-Tool main window. Open the movie by simply clicking on it.

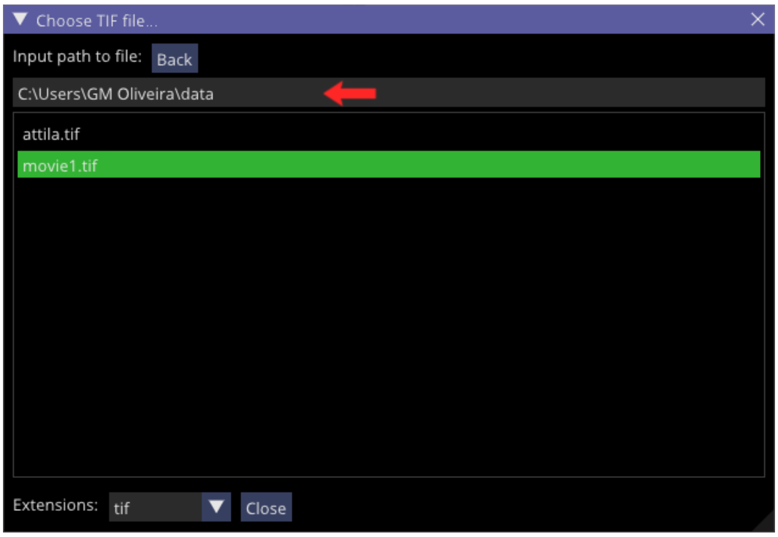

Loading the movie might take several seconds depending on size, compression and computer in use. Once the movie is finally loaded, the user will be presented with an interactive view port, where we can zoom in or out and move the image around. Similarly, the properties panel will display basic metadata if available. These are movie name, acquisition date, number of bits, image height and width, the number of frames, calibration from pixels into pertinent units and the name of each channel. We also have a slider from which we can change the frame displayed in the view port.

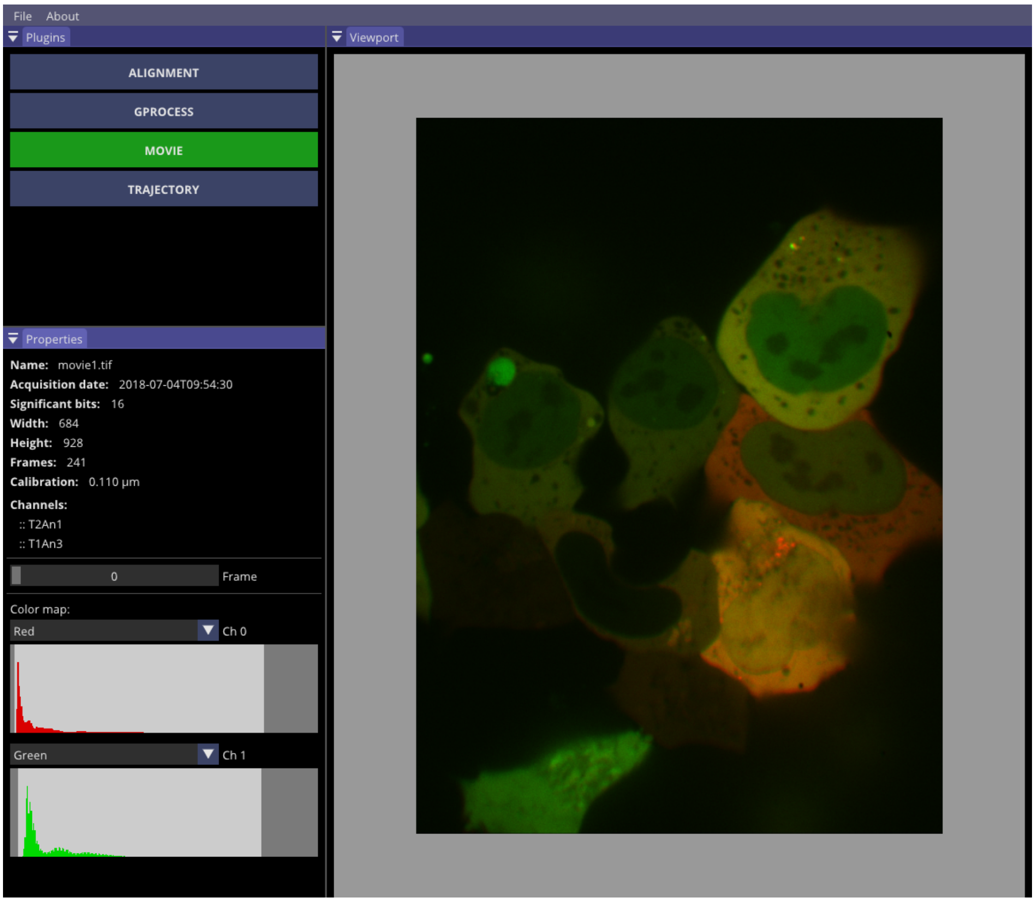

The color map allows the user to choose from a selection of colors, which look-up table will be use for each channel. Differently, we also can opt for none, hence hiding the corresponding channel. Finally, by dragging darker rectangles in either side of histogram widgets, we can set different and channel independent contrast.

Trajectory

Once the movie was imported, the user is able to load tracked trajectories into GP-Tool. There are 3 possible ways to do so:

1. Clicking on *File > Load trajectories...*;
2. Selecting Trajectory plugin under plugins tab and the "Load" button;
3. Key combination "Ctrl + T".

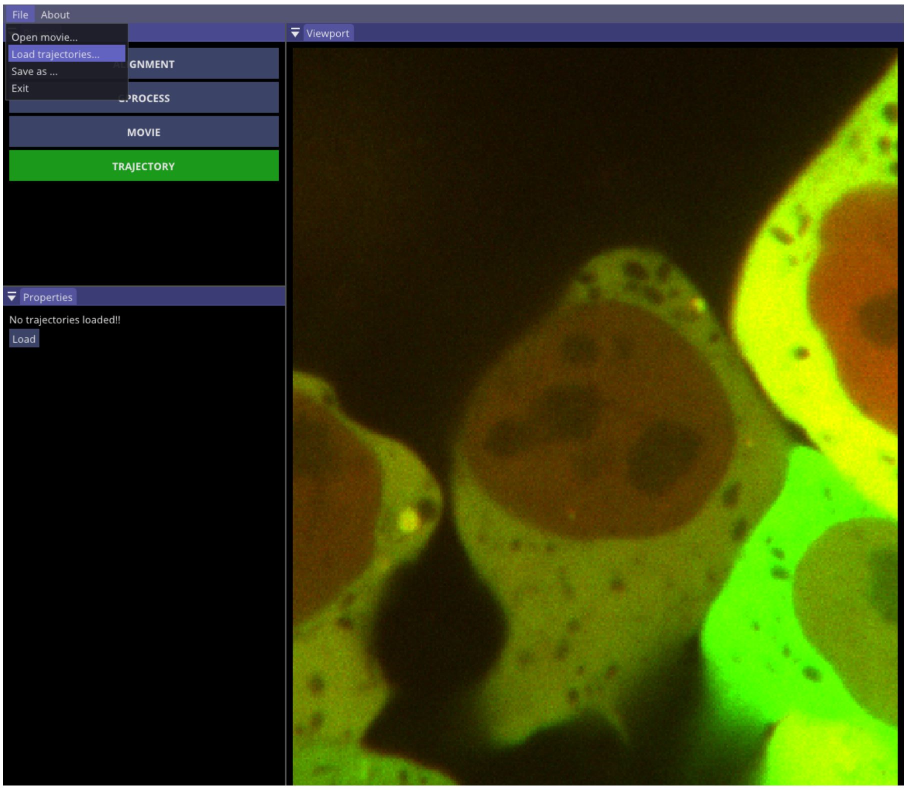

Any of these options will open a dialog where the user will be able to load a track file per channel. At this point, the user should also set an average size expected for spots. This value will be later used to enhance localization and estimate positional errors among other properties for each spot. If, for any reason, the user wants to change this value, there is the possibility to reload trajectories with a different expected size from within the plugin's properties tab.

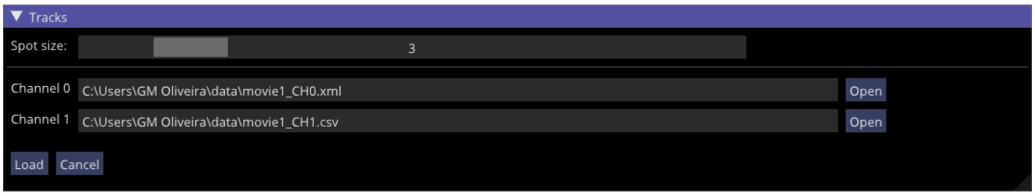

By default, GP-Tool accepts XML track files from ICY software or "handmade" CSV files. The following image shows how these files should resemble.

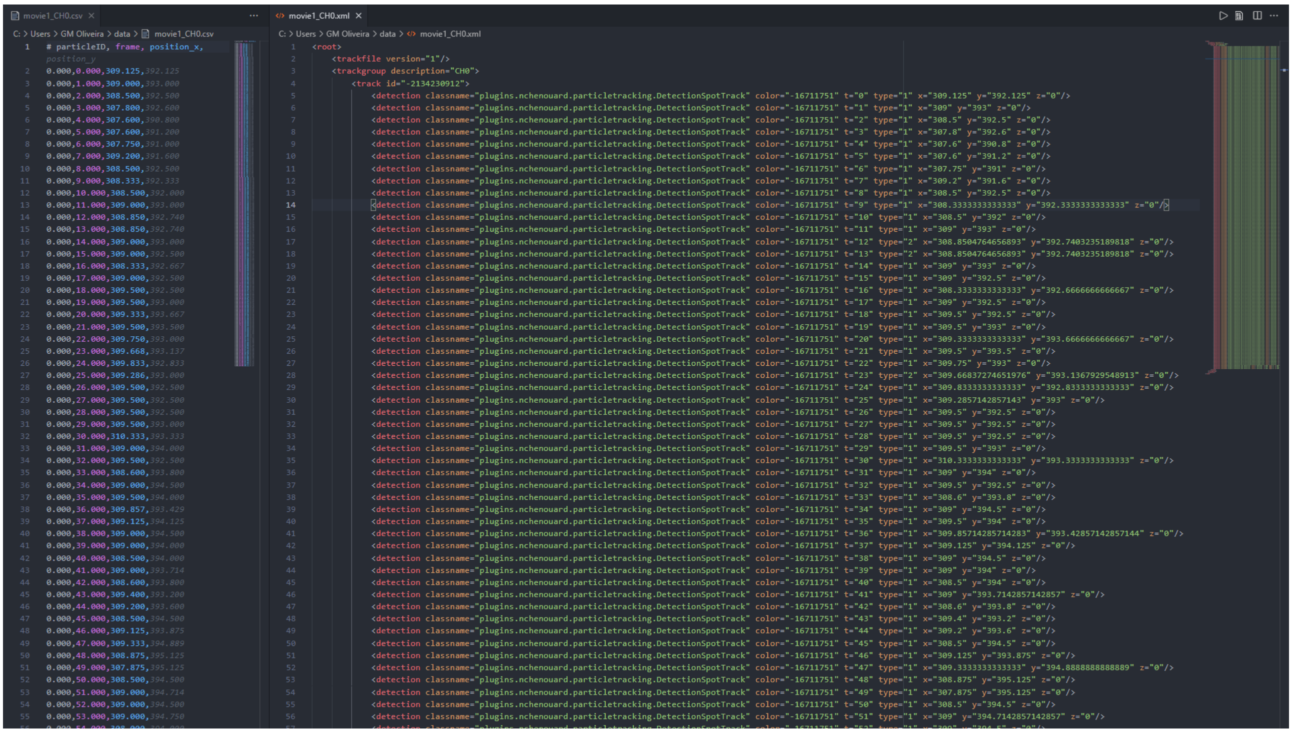

By clicking on load, GP-Tool will launch a set of algorithms to characterize each spot and remove possible outliers, such as false positives. Upon completion, the properties panel will open a tab for each channel in which all trajectories should have an unique color code assigned. The same color will be used to circulate corresponding spot in the view port if spot is checked.

The user can check further details about the trajectory described by each spot clicking in the button "detail". This option will open a tab containing information about detected frames, corresponding elapsed times in seconds (if metadata is properly configured), position in X and Y, localization error in X and Y, spot size in X and Y, background and above background signal. All coordinates, errors and sizes are given in pixels.

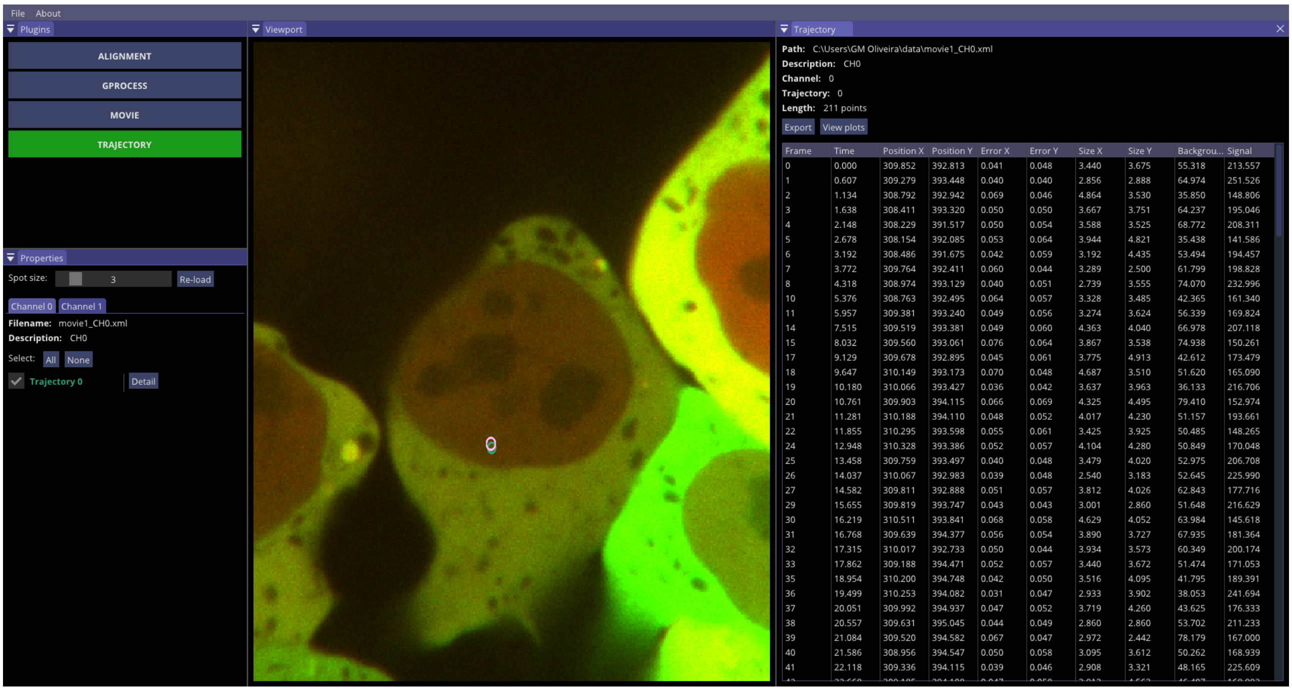

Aiming for a more intuitive analysis, clicking in "View plots" on top of the table, the user will be presented with plots containing information about the spot's position over time with a

95% credible interval, spot size and signal as follows

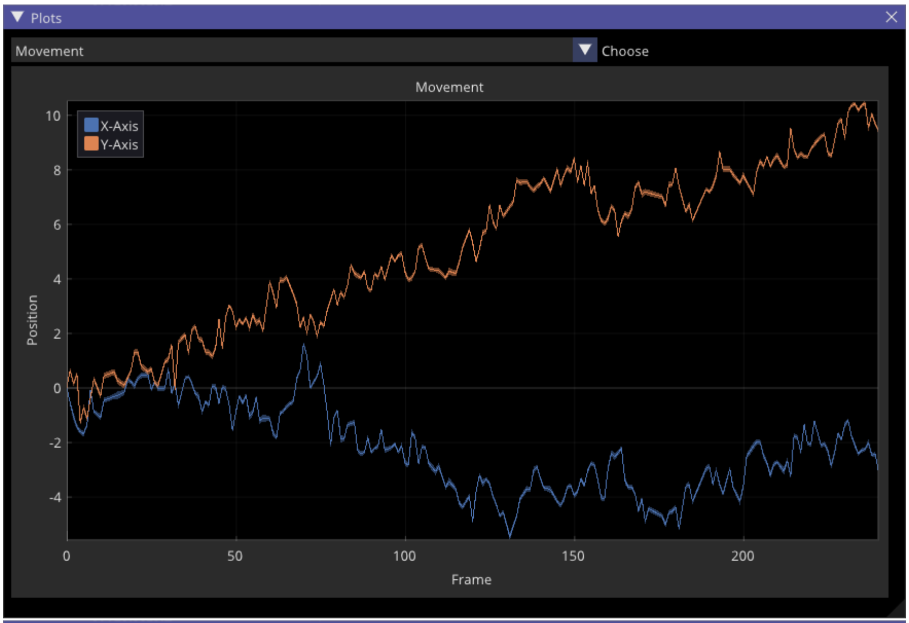

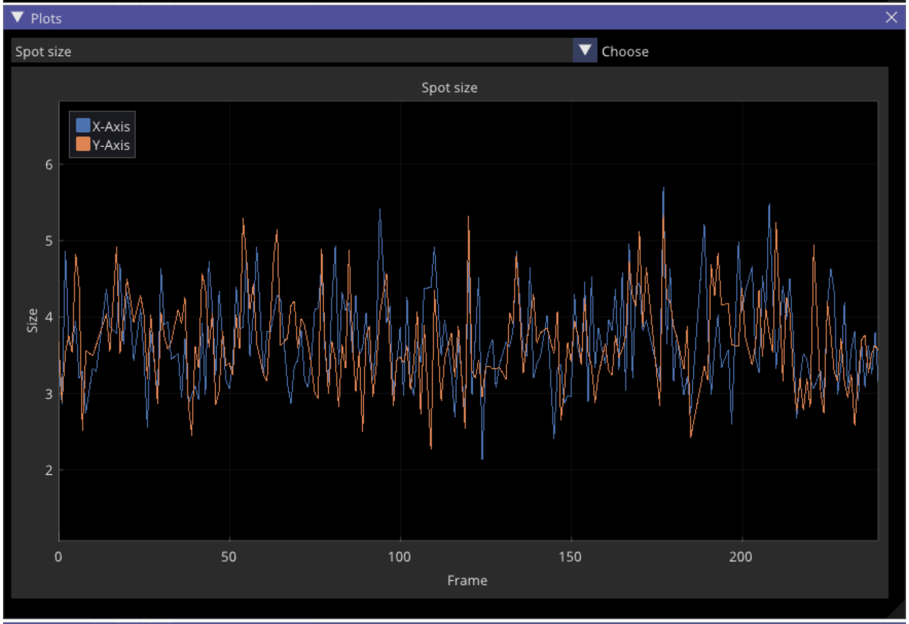

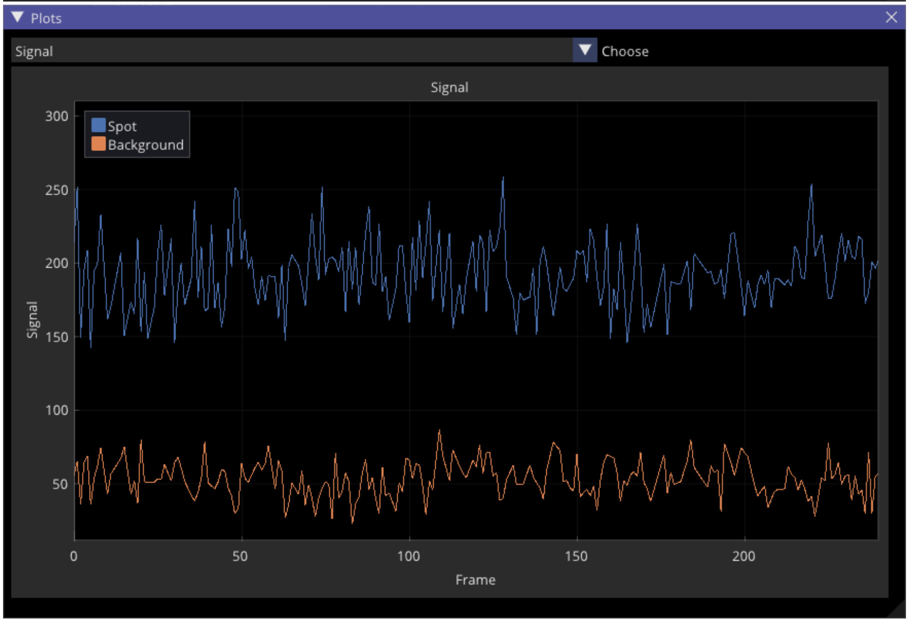

Finally, the user can choose to save this table in a CSV file by clicking on the "Export" button, which will open a save dialog.

G-Process

After all trajectories are imported, it is time to determine the diffusion properties of spots. The user has an option to analyze spots individually, by selecting one spot at the time under Trajectory plugin or to group spots. Once grouped, GP-Tool will assume that all selected spots are under a similar context, hence subject to similar background movement. In that sense, we could, for example, select all the spots in the nucleus of a cell and, like so, filter out the cellular movement from the apparent diffusion and anomalous coefficients. Differently, by selecting two or more spots close to fluctuating membrane, the dynamics properties inferred will attempt to filter out said fluctuations. In the next image, we first group both spots for a first analysis and, later, treat them independently. Comparing the results, we can observe how background movement affects inferred values.

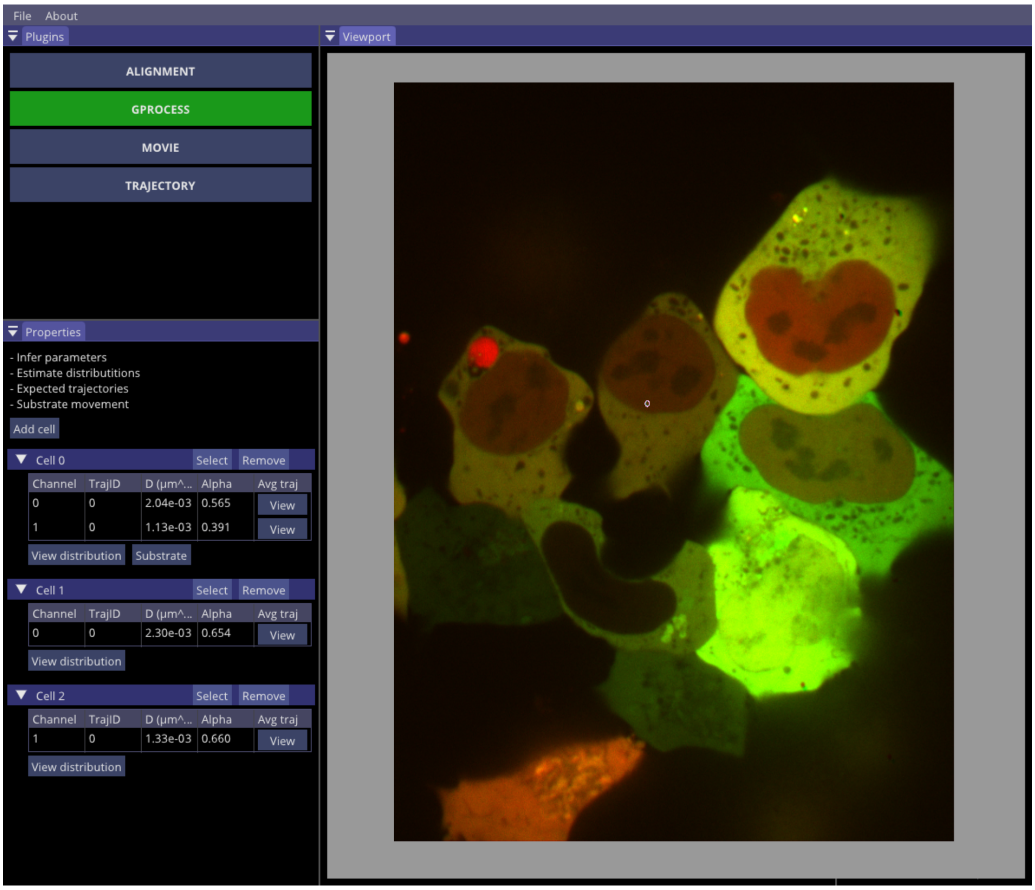

Evidently, by clicking on the button "Substrate" we are presented with a series of data estimated for the substrate from selected group of trajectories. In the substrate tab, we have inferred apparent diffusion and anomalous coefficients for the substrate as well as a probability density plot for these parameters. Additionally, GP-FBM will provide an estimation for the trajectory described by the substrate in the form of a table. The user can save this table by clicking in the button "Export" or visualize the data with "View plot", where the trajectory will be displayed with a 95% credible interval.

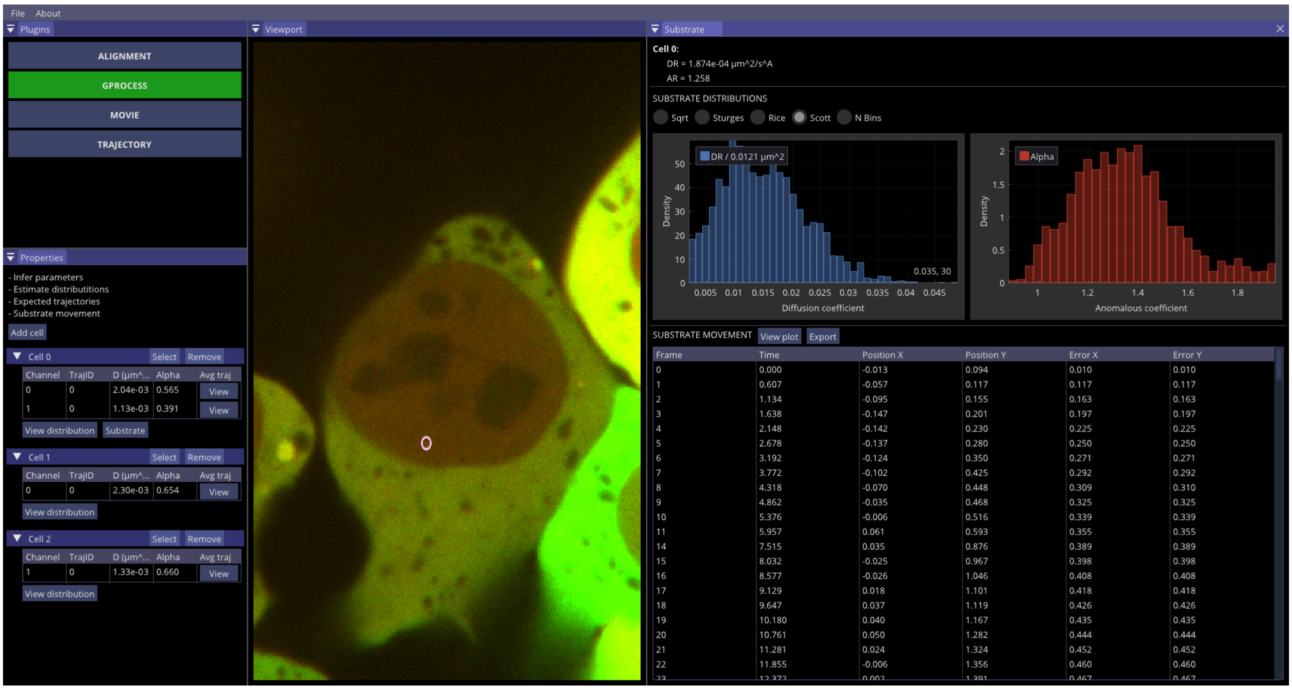

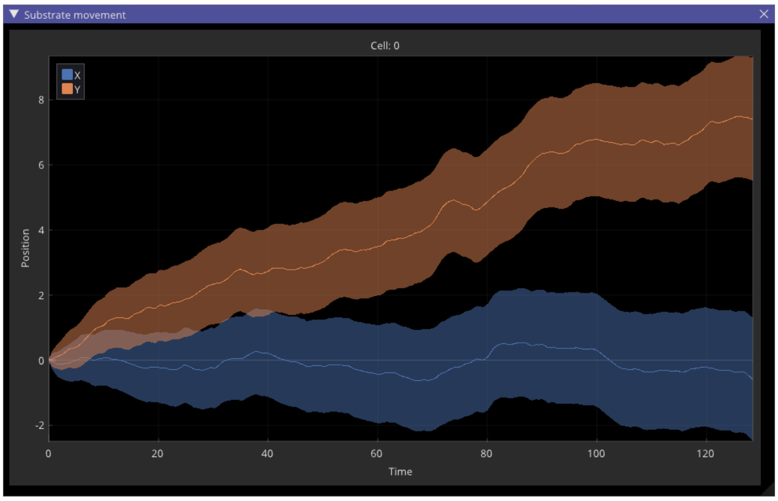

As an additional benefit of Gaussian processes, we can use measured spot locations and estimated dynamics parameters to interpolate the position of spots in time within a credible interval. GP-Tool allows the user to observe these curves using button "View" at the column "Average trajectory" in the properties panel. In the following image, points represent measured positions whilst continuous lines and shaded areas correspond to most probable trajectory in time with a 95% credible interval.

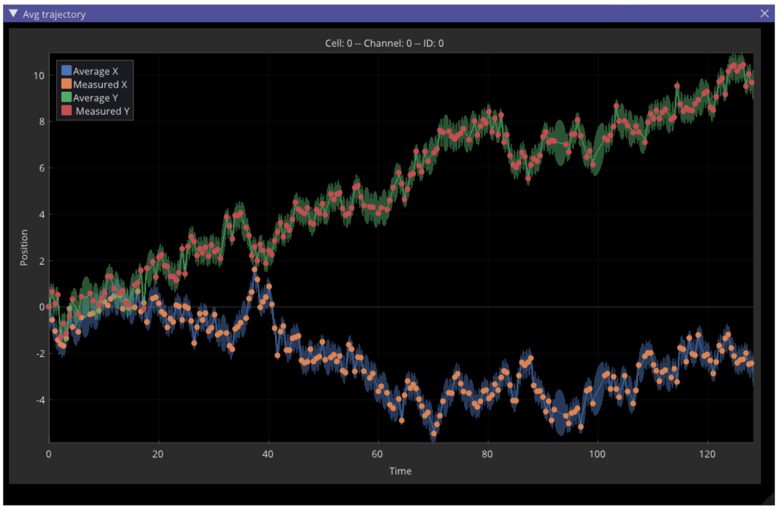

Finally, to determine how stable the inference of apparent diffusion and anomalous coefficients are, the user can consult the probability density function of each parameter estimated via Metropolis-Hastings clicking in the button "View distribution". For convenience, several standard binning methods are provided. Differently, the user can manually select any given number of bins.

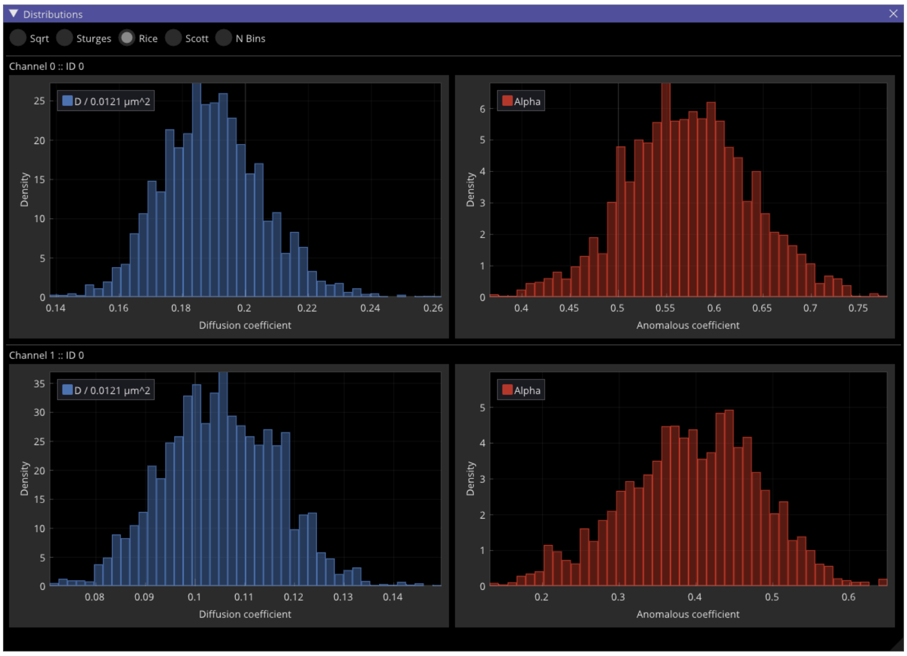

Alignment

Even though channel alignment is not strictly necessary for inference of diffusion properties as fractional Brownian motion is orientation independent and chromatic aberration is negligible for short trajectories, it becomes relevant once we are interested in the average distance between spots. For that reason, GP-Tool includes a plugin that will attempt to calculate a matrix based on generic affine transformations that will match common shapes in both channels. In the left most of the following images, we present a movie in which the dual-camera setup was badly calibrated. On the right, we present the results of an automatic alignment. Differently, the user might setup parameters manually.

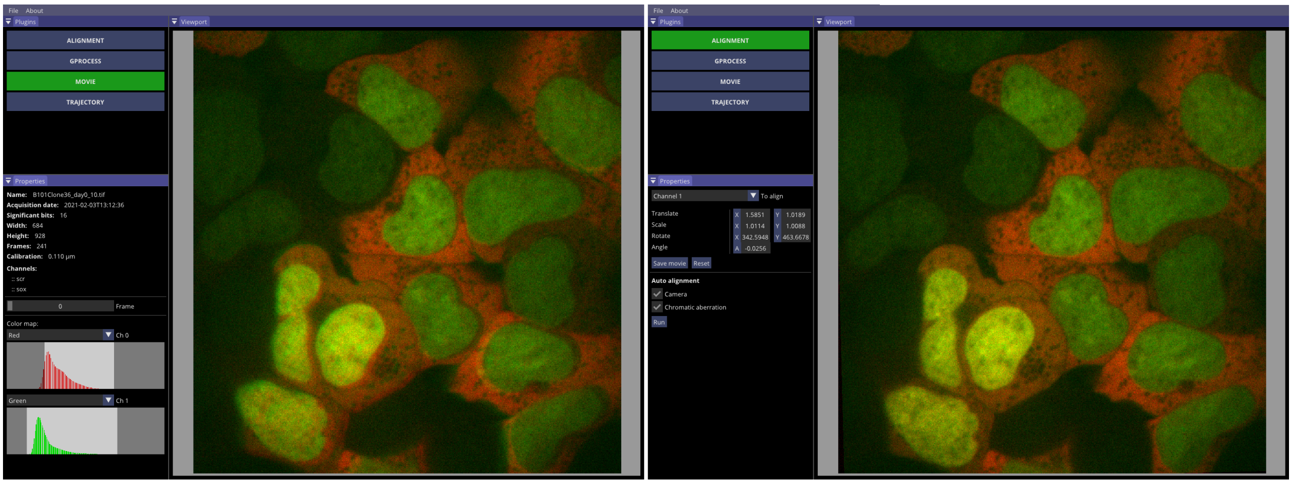

The plugin provides two types of auto-correction aiming for different types of errors. The first will combine translations and rotations in the attempt to correct a system with multiple badly calibrated cameras. Evidently, this type of correction is not necessary in the case of a setup with a single camera. On the other hand, any experimental protocol using multiple wavelengths is subjected to chromatic aberration. Its effect is not so relevant at image's center, but it might become non-negligible towards its edges.

Finally, the user might opt to save a new TIF-file with aligned channels. We can do that by clicking in button "Save movie".

Saving results

Once the analysis is complete, the user can save results by navigating to *File > Save as...* or the key combination "Ctrl + S". By its flexibility, "ease-of-use" and wide range of programming languages that can parse it, the JSON format was chosen. The final file will look as in the next image.

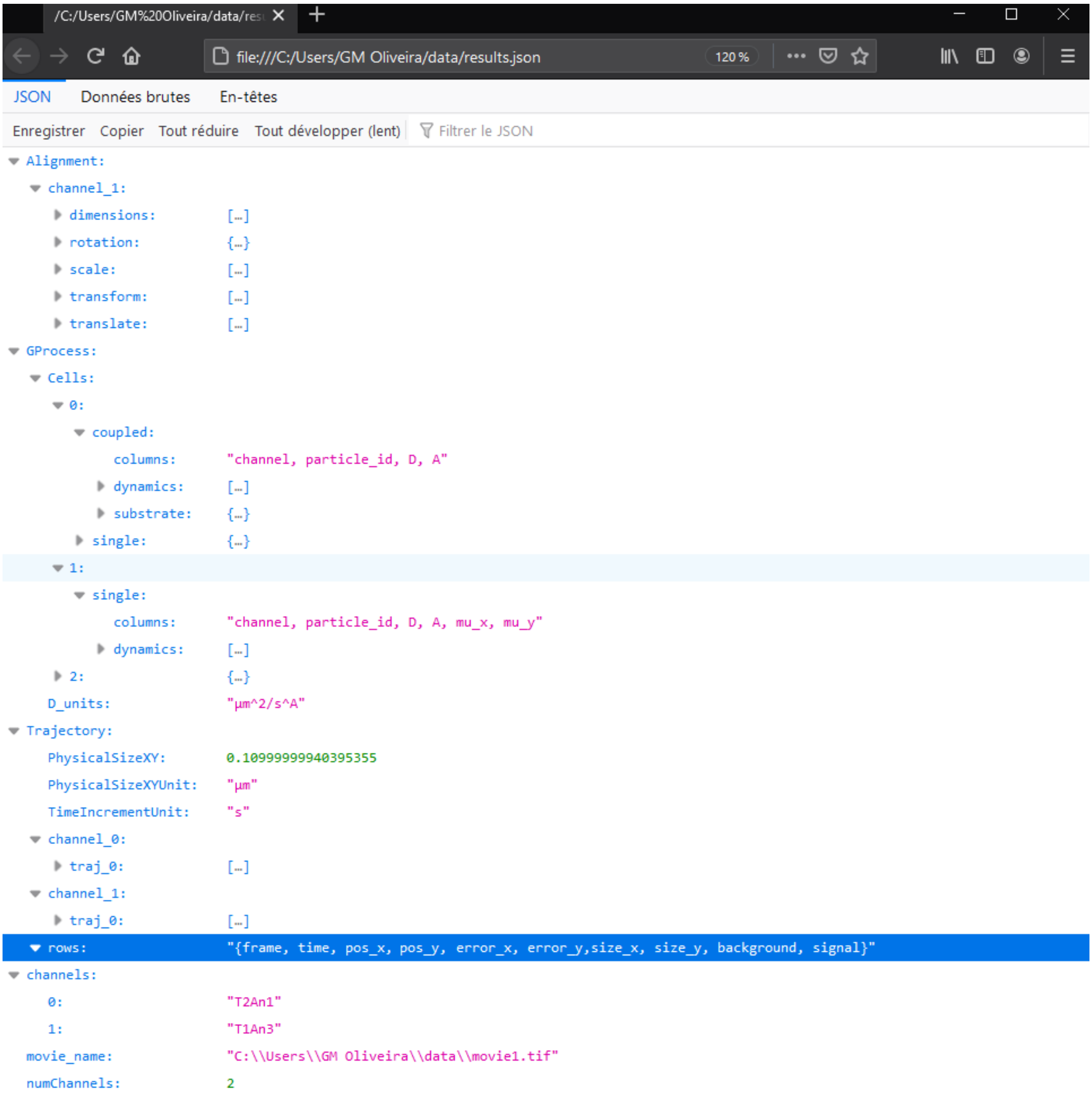

In a hierarchical organization, the file will show the original movie name and basic metadata on which the analysis was made. It will present enhanced non-aligned trajectories per channel (the contents of each row are identified) and inferred diffusion parameters. GProcess will always present results determined from individual trajectories. In cells (or groups) containing multiple spots, the coupled analysis will be provided along with substrate data. Finally, the alignment plugin will save its own data, that is, image dimensions and optimal translation, scaling and rotation parameters. For convenience, a ready-to-use transformation matrix is also provided. This matrix can be directly used to alignment trajectories if necessary.

Batching

In order to facilitate the analysis of hundreds of movies at a time, the core functions available in GP-Tool are wrapped in shared C++ libraries that can be linked to any C++ code. Depending on public demand, I might write python bindings for these libraries in the future. A template C++ program is provided in the GitHub repository along with a "CMakeLists.txt" which the user might use to compile its own program. I don't propose any standard mechanism to save output data as different types of analysis require alternative results to be saved. That said, popular formats I frequently use are HDF5, JSON and GDM (my own creation and also available in my GitHub page).

For the sake of efficiency in the case of Windows users, I suggest installing WSL2. On my personal computer, I got an improvement of about 40%in terms of speed.
