# Supplementary material for: Precise measurements of chromatin diffusion dynamics by modeling using Gaussian processes
Source: Nat Commun. 2021 Oct 26;12:6184. doi: 10.1038/s41467-021-26466-7 (PMC8548522; doi:10.1038/s41467-021-26466-7)
Supplement: Supplementary file 3 — Reporting Summary [file 41467_2021_26466_MOESM3_ESM.pdf]

nature research

Corresponding author(s): Nacho Molina, Thomas Sexton and Guilherme M. Oliveira

Last updated by author(s): Sep 30, 2021

Reporting Summary

Nature Research wishes to improve the reproducibility of the work that we publish. This form provides structure for consistency and transparency in reporting. For further information on Nature Research policies, see our Editorial Policies and the Editorial Policy Checklist.

Statistics

For all statistical analyses, confirm that the following items are present in the figure legend, table legend, main text, or Methods section.

n/a	Confirmed	
☐	☒	The exact sample size (*n*) for each experimental group/condition, given as a discrete number and unit of measurement
☒	☐	A statement on whether measurements were taken from distinct samples or whether the same sample was measured repeatedly
☐	☒	The statistical test(s) used AND whether they are one- or two-sided<br>*Only common tests should be described solely by name; describe more complex techniques in the Methods section.*
☐	☒	A description of all covariates tested
☒	☐	A description of any assumptions or corrections, such as tests of normality and adjustment for multiple comparisons
☐	☒	A full description of the statistical parameters including central tendency (e.g. means) or other basic estimates (e.g. regression coefficient) AND variation (e.g. standard deviation) or associated estimates of uncertainty (e.g. confidence intervals)
☐	☒	For null hypothesis testing, the test statistic (e.g. *F*, *t*, *r*) with confidence intervals, effect sizes, degrees of freedom and *P* value noted<br>*Give P values as exact values whenever suitable.*
☐	☒	For Bayesian analysis, information on the choice of priors and Markov chain Monte Carlo settings
☐	☒	For hierarchical and complex designs, identification of the appropriate level for tests and full reporting of outcomes
☒	☐	Estimates of effect sizes (e.g. Cohen's *d*, Pearson's *r*), indicating how they were calculated

Our web collection on statistics for biologists contains articles on many of the points above.

Software and code

Policy information about availability of computer code

Data collection	MetaMorph (version 7.0) https://www.moleculardevices.com/
Data analysis	ICY (http://icy.bioimageanalysis.org/); ImageJ (https://imagej.net/); GP-Tool (https://github.com/guilmont/GP-Tool)

For manuscripts utilizing custom algorithms or software that are central to the research but not yet described in published literature, software must be made available to editors and reviewers. We strongly encourage code deposition in a community repository (e.g. GitHub). See the Nature Research guidelines for submitting code & software for further information.

Data

Policy information about availability of data

All manuscripts must include a data availability statement. This statement should provide the following information, where applicable:

- Accession codes, unique identifiers, or web links for publicly available datasets
- A list of figures that have associated raw data
- A description of any restrictions on data availability

Microscopy data with analysis files that support the findings of this study have been deposited in Zenodo and can be accessed with 10.5281/zenodo.5359893, 10.5281/zenodo.5360028 and 10.5281/zen- odo.5361054.

ES Hi-C sequence data from [4] were taken from Gene Expression Omnibus (GSE96107), and mapped to mm9 and normalized with FAN-C [26]. The normalized submatrix (chr6:51500000-53000000) was then extracted for visualization. ES and neuronal precursor cell H3K27ac and CTCF ChIP-seq data were taken as bigWig files from Gene Expression Omnibus; GSE96107 for all except ES H3K27ac, taken from GSE49847) and visualized in R using the package rtracklayer.

Field-specific reporting

Please select the one below that is the best fit for your research. If you are not sure, read the appropriate sections before making your selection.

☒ Life sciences ☐ Behavioural & social sciences ☐ Ecological, evolutionary & environmental sciences

For a reference copy of the document with all sections, see nature.com/documents/nr-reporting-summary-flat.pdf

Life sciences study design

All studies must disclose on these points even when the disclosure is negative.

Sample size	Sample sizes were not determined in advance and instead were defined by the technical limitations of this study.
Data exclusions	We excluded cells with less than 2 spots as our method requires at least two spots to correct for substrate movement. We also excluded cells with sopt trajectories shorter than 100 timepoints because the inference error increases with short trajectories as shown with simulated data.
Replication	All attempts of replication were successful.
Randomization	Not performed as this is not relevant for this study.
Blinding	Not performed as this is not relevant for this study.

Reporting for specific materials, systems and methods

We require information from authors about some types of materials, experimental systems and methods used in many studies. Here, indicate whether each material, system or method listed is relevant to your study. If you are not sure if a list item applies to your research, read the appropriate section before selecting a response.

Materials & experimental systems

n/a	Involved in the study	
☒	☐	Antibodies
☐	☒	Eukaryotic cell lines
☒	☐	Palaeontology and archaeology
☒	☐	Animals and other organisms
☒	☐	Human research participants
☒	☐	Clinical data
☒	☐	Dual use research of concern

Methods

n/a	Involved in the study	
☒	☐	ChIP-seq
☒	☐	Flow cytometry
☒	☐	MRI-based neuroimaging

Eukaryotic cell lines

Policy information about cell lines

Cell line source(s)	ANCHOR cell lines derived from the J1 mouse embryonic stem cell line (ATCC SCRC-1010). The TetR::GFP cell line is derived from PGK12.1 mouse ES line and was kindly provided by Dr. Luca Giorgetti (FMI, Basel).
Authentication	ES state of cells are periodically verified by qRT-PCR for known pluripotency markers and colony morphology.
Mycoplasma contamination	All cell lines tested negative for mycoplasma contamination.
Commonly misidentified lines (See ICLAC register)	No commonly misidentified cell lines were used in the study.
